# Mask-Wearing Behaviors after Two Years of Wearing Masks Due to COVID-19 in Korea: A Cross-Sectional Study

**DOI:** 10.3390/ijerph192214940

**Published:** 2022-11-13

**Authors:** Miji Kwon, Wonyoung Yang

**Affiliations:** 1 Department of Speech-Language Rehabilitation & Counseling, Gwangju University, Gwangju 61743, Korea; 2 Division of Architecture, Gwangju University, Gwangju 61743, Korea

**Keywords:** COVID-19, face masks, mask-wearing behaviors, questionnaire, discomfort, anxiety, Korea

## Abstract

In Korea, wearing masks in public places has become the norm during the prolonged coronavirus disease 2019 (COVID-19) pandemic. This cross-sectional study investigated the mask-wearing behavior of Koreans (*n* = 433) via online mode living in Seoul and Gwangju after wearing a mask in public spaces for two years due to COVID-19. The respondents selected their face masks based on season, gender, age, occupation, mask-wearing hours, mask filter performance, mask shape, and mask color. The general discomfort caused by wearing a mask was divided into physical and speech discomfort, and it was not correlated with anxiety when not wearing a face mask. Speech discomfort caused by wearing a mask was correlated with general discomfort, clear speech, vocal pain, anxiety, and only-indoor mask-off plans. Anxiety when not wearing a mask appeared to affect both indoor and outdoor mask-off plans. The more uncomfortable and less anxious respondents were when not wearing a mask, the sooner they wanted to discontinue wearing masks indoors and outdoors. It is expected that the use of masks will continue in the future and that there may be differences in the place and time of use of masks in Korea and around the world due to new infectious diseases and fine dust. Facial masks can be worn more comfortably and conveniently if the discomfort and anxiety of wearing a mask are improved by considering various behaviors when wearing a mask in the future.

## 1. Introduction

Since the World Health Organization (WHO) declared the coronavirus disease 2019 (COVID-19) a pandemic on 11 March 2020 [1], wearing masks in public places has become the norm to reduce the spread of the virus in Korea. On 13 November 2020, wearing face masks became compulsory in public places [2]. After overcoming the difficulties of COVID-19, on 2 May 2022, wearing masks outdoors became voluntary rather than mandatory [3]; however, many people have continued to do so.

Korea has experienced several large outbreaks during the past two years and has been able to flatten the epidemic curve without lockdown [4,5]. The containment of COVID-19 in Korea was successfully achieved without an economic blockade because Koreans actively followed quarantine guidelines [4,6,7]. Face masks and hand sanitizers have become prerequisites in public spaces. Even before the mandatory mask-wearing policy was implemented in August 2020, 90.8% of people in supermarkets, 91.8% of people in underground shopping malls, and 83.6% of people on the street wore masks properly [8].

The mask-wearing behaviors of Koreans have been intensively investigated during the COVID-19 pandemic. Chang et al. [9] conducted a cross-cultural comparison study between the United States (*n* = 150) and Korea (*n* = 150) and found that Americans had to be encouraged to try wearing masks on their own, while Koreans considered the social benefits of wearing masks. Chung et al. [10] investigated the mask-wearing behavior of Koreans based on theories of individualism and collectivism. Two online surveys were conducted on 9 July 2020 (*n* = 1000) and 21–29 December 2020 (*n* = 1569). They concluded that the general collectivism of the Korean people was mutually synergetic with horizontal individualism in responding to COVID-19 in Korea. Mo and Park [11] found that horizontal, vertical, cognitive, and affective we-ness positively influenced mask-wearing behavior perception in their survey study (*n* = 720). Using an online survey, Kim and Han [12] reported that mask use was expected to increase further if people perceived a personal need to wear masks, if their peers perceived the importance of mask use, and if they possessed civic consciousness that considered society as a whole (*n* = 280). Han et al. [13] investigated explicit and implicit attitudes toward mask-wearing among 70 undergraduate and graduate students in Korea. The results revealed that respondents explicitly and implicitly perceived mask-wearing as safe, and that the two attitudes were significantly correlated. Unlike the attitudes toward mask-wearing, however, respondents still associated faces covered by a rectangular, black object with a threat. Han et al. [13] found that the motive for disease avoidance was positively related to both explicit and implicit attitudes toward mask-wearing.

Complications with face masks lead to an increased risk of aspiration, difficulty in delivering a fraction of inspired oxygen, painful facial trauma, skin changes, ear pain due to the elastic straps of the mask, difficulty in expectoration, claustrophobia, and so on [14,15,16,17]. Furthermore, face masks degrade speech recognition [18,19,20,21,22,23,24] and block facial communication [25,26,27,28,29,30]. The audiovisual speech recognition performance of transparent face masks has been studied [25,26,27,28,29]. In the past two years, there has been an increasing demand in Korea for masks that are safer, more convenient, and that can reveal individuality. Thus, face masks have transformed from personal health products to social epidemic prevention products [31].

However, research on the optimal face mask for long-term wear is still in its early stages of development. A few Asian research groups have begun to work on improving face masks. In September 2020, six months after the pandemic was declared by the WHO, Ma and Kim [31] published the results of a survey conducted with 322 valid specimens regarding the tendency to choose when purchasing a mask in Korea and China. They emphasized user-centered design, considering the influence of gender and age, while proposing to consider commercialization, individualization, and fashion in the design of the face mask. Occupational factors have very little influence on mask design, and the influence of gender and age on it needs to be studied in depth [31]. Liu et al. [32] developed a reusable face mask through a systematic method that follows a modular design concept based on industrial design in Taiwan. Ipaki et al. [33] performed usability testing of two types of face masks in Iran and developed a concept design of a face mask with anthropometric support and the face forms of different users for a suitable respiratory protective cover against COVID-19. Seo and Lee [34] showed that the mask design element had a positive reinforcing effect on a user’s image, increased product purchase reliability when purchasing a mask, and affected satisfaction when wearing a mask. Palcu et al. [35] suggested that linking facial mask-wearing to an individual’s identity is a promising strategy to encourage mask-wearing.

The purpose of this cross-sectional study was to investigate the mask-wearing behavior of Koreans after wearing a mask in public spaces for two years from the end users’ perspective due to the COVID-19 pandemic. Public use of face masks should be taken as a precautionary measure in the long-term COVID-19 era with the expected multiple waves of infection. In this study, the preference for mask types, inconveniences due to mask-wearing, and future plans for mask-wearing were investigated as preliminary data for various disciplines. If it is necessary to wear a mask daily, it would be effective to identify and improve various inconveniences, including speech recognition, to allow people to wear a comfortable and convenient mask.

## 2. Methods

### 2.1. Questionnaire Design

Previous surveys [9,36,37,38] on mask-wearing behavioral responses to COVID-19 were reviewed, and the authors included additional questions related to face masks. A total of 18 questions were developed, including 3 questions on general information about gender, age, and occupation and 15 questions on mask-wearing behaviors. The structured questionnaire consisted of questions covering several areas: (1) daily choice of face mask, (2) discomfort due to a face mask, (3) anxiety due to not wearing a mask, and (4) future plans for mask-wearing. In addition, the questions were structured such that duplicate answers were possible for the reasons for discomfort in wearing a mask, discomfort when talking while wearing a mask, and anxiety when not wearing a mask. Other items were added to allow the respondents to express their opinions on answers that were not included in the questionnaire.

The multiple choices of mask shape and color were based on two previous studies. Koreans prefer pleated surgical, vertical folding (VF), and horizontal folding (HF) types of masks [31,34]. For mask colors, white, black, blue, and green were preferred in general [31]. Multiple choices for mask filter performance were selected in consideration of popular preference based on Korean filter (KF) standards, KF-AD (anti-droplet), KF-80, KF-94, and regular fabric masks. KF masks, classified as health masks, are intended to prevent the passage of particulate matter (PM), such as yellow dust, and are certified by the Korea Ministry of Food and Drug Safety. The number next to the KF mark indicates the percentage of particles that the mask can prevent from passing through [39]. A KF-94 mask is equivalent to the N-95 mask [40]. A fabric mask, which has diverse designs, is more flexible and air permeable than the other masks, and there are no specific market requirements to sell it. Anti-droplet masks are generally light, air permeable, and effective in blocking droplet transmission. The filter performance of pleated surgical masks was equivalent to that of KF-AD masks. The seven types of masks in the questionnaire included a combination of mask shape and filter performance: KF-AD VF, KF-AD HF, surgical, KF-80 VF, KF-80 HF, KF-94 VF, and KF-94 HF.

A panel of three experts (professor of speech, language, and hearing science; professor of health and welfare; and psychotherapist) evaluated the draft questionnaire as to whether the questions were suitable for the general public according to the purpose of the study and whether multiple choices were effective in deriving meaningful results. Before the online survey, a pilot survey was conducted with six ordinary people—two for each age group—to understand the applicability of the questionnaire and survey tool, in which no problems emerged.

### 2.2. Participants and On-Line Survey

A cross-sectional survey design was adopted to evaluate mask-wearing behaviors during the COVID-19 pandemic using an anonymous online questionnaire distributed in Seoul and Gwangju, Korea. Korean residents aged ≥18 years were recruited for this study. Potential respondents were invited to participate via e-mail and text messages and were able to access the questionnaire through a URL link. Owing to the form of recruitment, it was not possible to calculate the survey response rate. Data were collected on 22–30 March 2022 via convenience sampling. A total of 445 respondents completed the survey, and 433 (97.36%) were included in the analysis. The respondents could only continue with the survey if they stated that they had given their consent and read the terms and conditions. The Gwangju University approved the survey and consent to participate.

The survey data were analyzed using IBM SPSS Statistics 27 (IBM, Armonk, NY, USA). Descriptive statistics and a chi-squared test were used to examine the responses to the questions. Spearman’s rank correlation coefficients were calculated for all 18 questions answered. Statistical significance was set at *p* < 0.05.

## 3. Results

### 3.1. Demographic Information

A total of 433 respondents between the ages of 18 and 80’s were analyzed. Three demographic questions were used as factors for further analysis: gender, age, and occupation. Table 1 lists respondents’ demographic characteristics.

### 3.2. Overall Results

Table 2 lists respondents’ daily choice of face mask. One-third of the respondents (31.6%) wore masks for six to nine hours a day; one-fifth (21.0%) wore masks for nine to 12 h a day, and most of them (82.7%) answered that they preferred KF-94 masks. The preference for the mask shape was not clearly distinguished, but the preferred mask color was predominantly white. The pleated surgical mask was popular in summer (23.6%), but in winter only 5.8% of respondents preferred the pleated surgical mask. Although 82.7% of the respondents preferred the KF-94 mask (HF + VF) in general, only 60.7% of the respondents (winter) used the KF-94 mask (HF + VF) the most in the seasonal mask selection.

Table 3 lists the responses to discomfort caused by wearing face masks. In general, 84.5% of the respondents answered that they felt “moderately uncomfortable” to “strongly uncomfortable” when wearing a mask. The causes of discomfort were “shortness of breath” (25.5%), “ear pain due to the mask strap” (22.8%), “difficulty speaking” (22.2%), and “mask movement when speaking” (18.8%). When talking while wearing a mask, 85.4% of the respondents answered that they felt “moderately uncomfortable” to “strongly uncomfortable”. When talking while wearing a mask, the causes of discomfort were “cannot hear well” (36.5%), “speak loudly” (30.9%), and “speak repeatedly” (28.0%). When speaking while wearing a mask, 81.0% of the respondents answered that their pronunciation was “moderately clear” to “strongly clear”. When speaking while wearing a mask, 50.6% of respondents felt vocal pain “moderately” to “strongly”.

Table 4 lists the anxiety due to not wearing a mask and the future plans for mask-wearing. When the respondents did not wear a mask, 69.7% of them answered that they felt anxiety “moderately” to “strongly”. The anxiety was mostly caused by the increased risk of droplet transmission of COVID-19 (50.2%). However, the respondents considered the negative social pressure of not wearing a mask (29.4%). In addition, 18.8% of respondents cited psychological anxiety when not wearing a mask, which was caused by the habit of wearing a mask over the past two years.

If the expert group declared the end of COVID-19, one-third (32.1%) said they would not wear a mask outdoors, and 27.5% said they would not wear a mask indoors. Almost one-third answered that they would not wear a mask outdoors or indoors within three months if the expert group declared the end of COVID-19. Even if the expert group declared the end of COVID-19, 13.9% (outdoors) and 14.5% (indoors) said they would continue to wear masks for more than a year.

Spearman’s rank correlations were used to understand the correlations among the items asked, as listed in Table 5. The respondents’ ages and occupations were highly correlated in this study, which is expected. Mask-wearing hours were correlated with age and occupation. Seasonal mask choice was correlated with gender, age, occupation, mask-wearing hours, mask filter performance, mask shape, and mask color. Gender was highly correlated with discomfort- and anxiety-related items.

General discomfort was correlated with speech discomfort, clear speech, vocal pain, and indoor/outdoor mask-off plans. Speech discomfort was correlated with general discomfort, clear speech, vocal pain, anxiety, and indoor mask-off plans. Anxiety correlated with clear speech, vocal pain, and indoor/outdoor mask-off plans (*p* < 0.0005). General discomfort was negatively correlated with indoor/outdoor mask-off plans, while anxiety when not wearing a mask was positively correlated with indoor/outdoor mask-off plans. Indoor and outdoor mask-off plans were strongly correlated (ρ = 0.777, *p* < 0.0005), and mask-off plans were correlated with summer mask choice.

### 3.3. Analysis by Age, Occupation, and Gender

A chi-squared test was used to test the probability of the independence of age, occupation, and gender (Table 6, Table 7 and Table 8). Age seemed to affect the daily choice of face mask. Occupation affected the daily choice of face mask, discomfort, anxiety, and future mask-wearing plans, which covered all the questions asked. The gender effect was found in discomfort, anxiety, and future mask-wearing plans rather than in the daily choice of face mask. The shape of the mask, overall degree of discomfort caused by wearing the mask, and effort to speak clearly while wearing the mask were found to be independent of age, occupation, and gender.

Among the three age groups, the two younger age groups (20’s–30’s and 40’s–50’s) exhibited similar trends. On the other hand, respondents older than 60 years showed different trends compared to the other two younger age groups. The number of respondents who wore masks for less than 3 h per day increased with age. Their mask-wearing duration was shorter, their preference for black masks was lower, and their preference for breathable mask filters, such as KF-AD and surgical masks, was higher. The proportion of respondents in their 60’s and older (15.5%) who felt very uncomfortable when speaking was half of that of those in their 20’s and 30’s (31.3%).

The seasonal mask choice was dependent on occupation. Almost 60% of the students wore KF-94 masks during the winter and spring/autumn. In summer, 51.4% of the students used the KF-AD and surgical masks; however, 42.5% still used the KF-94 masks. The homemaker and freelancer groups preferred KF-AD and surgical masks, regardless of the season. The group with the highest rate of feeling strong discomfort when speaking while wearing a mask was that of the students, but 30.9% reported no vocal pain. Half of the freelancers (51.7%) reported no vocal pain. A quarter of the student and freelancer groups reported no anxiety when not wearing a mask. Employees and homemakers showed higher anxiety rates than freelancers did. No difference among occupations was observed in the future outdoor mask-wearing plan, but the future indoor mask-wearing plan depended on occupation. A quarter of the homemakers showed a tendency to continue wearing masks for more than a year after the end of COVID-19, the highest percentage among all occupations. In contrast, 41.4% of freelancers said they would not wear a mask immediately after the end of the COVID-19 pandemic.

The percentage rate of no anxiety about not wearing a mask was higher among men than among women. The rate of those who would remove their mask immediately, both indoors and outdoors, at the end of COVID-19 was also higher in men than in women. The proportion of KF-AD users was also higher among men than among women.

### 3.4. Other Discomfort and Anxiety

Table 9 lists subjective responses to three questions (Q10, Q12, and Q16). Regarding discomfort when wearing a mask, many comments (*n* = 69) were made by the respondents. Facial discomfort, including sweat, moisture, skin disorders, and makeup smudging, was most frequently noted by the respondents. In addition to the four multiple choices of “shortness of breath”, “difficulty speaking”, “mask movement when speaking”, and “ear pain due to mask straps”; “foggy glasses” was the most frequently selected discomfort item.

Compared to general discomforts, discomforts when talking while wearing a mask other than the multiple choices of “cannot hear well”, “speak repeatedly”, and “speak loudly” were not frequently listed. Invisible mouth shape and facial expressions were considered speech discomfort. Mask movement, smell, and moisture inside the mask were uncomfortable when talking while wearing a mask.

Anxiety when not wearing a mask was not commented upon as many other reasons not presented in the survey, such as “increased risk of droplet transmission”, “negative public opinion on not wearing a mask”, and “psychological anxiety due to wearing habits for two years”.

## 4. Discussion

### 4.1. Differentiation of Mask-Wearing

Over the past two years of COVID-19, Koreans have become more interested in the issues of how and what kind of masks they are wearing, in addition to why they wear them [41]. Studies have reported on whether masks were worn [8] in the early stages of COVID-19 in 2020. Research on why masks are worn medically [39,40,42], socially [37,43,44], culturally [9,10,11], and psychologically [36] has been widely conducted. Recently, studies on face masks have been extended to include their personalization [31,34,45]. The present cross-sectional study captures the differentiation of mask-wearing in terms of color and filter performance.

Previous studies [31,34] have only found a general preference for mask shape, color, and filter performance without seasonal choice; and have shown that horizontal folding, vertical folding, and pleated masks were also common [31,34]. In their study, Seo and Lee [34] showed that the preference for the vertical foldable mask was lower than that for the horizontal foldable mask and pleated mask; however, the preference for the vertical foldable mask was almost similar to that of the horizontal foldable mask, and it was found to be higher than that of the pleated mask in this study.

The respondents wore different masks in each season. A pleated surgical mask has the lowest fit factor [46] and therefore a relatively high leakage rate, which makes breathing easier and less hot in summer. Respondents preferred a surgical mask during this season, although the filter performance was not significant. However, in the winter or spring/autumn seasons, a KF-94 mask was generally preferred, considering its filter performance. The seasonal mask choices differed according to age, occupation, and gender. In the summer, a surgical mask was generally preferred, while people in their 40’s to 50’s or employed still preferred to use a KF-94 mask. In the winter and spring/autumn seasons, younger people, students/employed, and women generally preferred a mask with a KF-94 filter, while people over 60, homemakers/freelancers, and men preferred a light mask with which it is relatively easy to breathe.

While white masks were predominant, younger respondents less than 60 years old also used black masks (more than 20%). However, people older than 60 years have been shown to prefer gray scale or chromatic masks to black ones.

### 4.2. Discomfort Due to Masks

The discomfort associated with wearing a mask was analyzed as physical and speech discomfort. Physical discomfort was generally referred to as discomfort when wearing a mask, and 84.5% of respondents complained of more than moderate discomfort. Before COVID-19, individuals in Korea chose to wear masks because of the presence of fine and yellow dust. However, as wearing a mask became a government-compulsive strategy, daily mask-wearing increased related discomfort in various aspects. Our results are consistent with those of previous studies, which have reported eyeglass fogging, moisture, and skin and makeup problems as the causes of discomfort [32,45,47], along with shortness of breath, difficulty speaking, ear pain, and movement of the mask while speaking [47]. In particular, 82.7% of the respondents in this study used KF-94 masks, a high-performance filter that caused difficulty breathing [45]. When mask-wearing continues and is prolonged, efforts to improve the comfort of wearing a mask have been reported. Ipaki et al. [33] proposed a new mask design concept considering anthropometric differences regarding 30 types of jaws for better fitting. Liu et al. [32] developed an innovative reusable mask based on technical measures. Chao [48] also proposed three mask designs: a mask with a water channel, folding pattern mask, and a mask combined with a cap rim. O’Connor et al. [15] designed a 3D-printed mask extender to relieve posterior auricular discomfort. Even mask retainers have been investigated for convenient and hygienic mask-wearing, and their demand and supply have increased with function and personality [45]. Skincare to minimize problems due to long-term mask-wearing has been studied [17,49,50].

Speech discomfort refers to discomfort when talking while wearing mask. It was affected by three demographic factors: age, gender, and occupation; 85.4% of the respondents complained of more than moderate discomfort when wearing one, which is similar to the results for physical discomfort in this study. This finding is consistent with those of previous studies. Face masks negatively impact verbal communication [51,52], and the effects of face masks on speech recognition have been investigated both physically and psychologically. Face masks mask the acoustic signals of speech, transform the speech frequency spectra, and physically attenuate the level of speech [19,22,23,25,53,54]. In noisy conditions, face masks adversely affect speech recognition [20,26,55,56]. Different types of masks generally yield similar accuracies with low levels of background noise, but differences between masks become more apparent with high levels of noise [57].

Vocal effort and vocal pain were other speech discomforts due to a face mask. In this study, 58.9% of respondents tried to speak loudly or repeatedly. When speaking while wearing a mask, 81.0% of respondents tried to enunciate in a more than moderately clear manner. Moreover, 50.6% of the respondents felt vocal pain more than moderately when talking while wearing a mask, which is supported by scientific evidence from previous studies. Speech while wearing a mask was rated as more effortful than unmasked speech, particularly with background noise [26]. The use of face masks increased the perception of vocal symptoms and discomfort, especially in individuals who wore them for professional and essential activities [58].

The use of face masks is a proven mitigation strategy to minimize the spread of COVID-19 and other airborne diseases. However, it may place individuals with hearing, speech, and language disorders at a greater risk for communication problems in their daily lives [59]. If wearing a mask is inevitable for a long time, it will be necessary to improve the mask by minimizing the detailed discomfort presented in Table 9.

### 4.3. Anxiety When Not Wearing a Mask

In general, half of the respondents were anxious or strongly anxious when they did not wear a mask. This is somewhat consistent with previous studies that found a positive correlation between face mask use and anxiety [37,60,61,62]. The causes of anxiety when not wearing a mask were medical, social, and psychological. Medical reasons were ranked first, followed by social and psychological ones.

By occupation, while anxiety rates for the employed and homemaker groups increased, those for the student and freelancer groups decreased. Anxiety when not wearing a mask was greater in women than in men, which seemed to affect when they intended to stop wearing a mask. No age differences were found in the anxiety levels in this study. This is inconsistent with Krishna et al. [60] who reported greater mask-related worry in older adults (≥60 years) than in younger adults (18–35 years) in Germany. This discrepancy might be caused by social and cultural differences in mask-wearing attitudes between Koreans and Germans [63,64].

The respondents made decisions based on medical facts combined with their own social and psychological aspects. It could be interpreted that medical professionals should provide the general public with accurate and objective information about the effects of wearing masks. The conceptualization of mask-wearing in Saint and Moscovitch’s review paper [61] regarding the potential effects of mask-wearing on social anxiety could be used as a potential safety behavior for people with higher levels of social anxiety. They suggested that it will likely be important for clinicians to explore whether and when their clients with social anxiety choose to wear masks and for what reasons. As we transition into the post-pandemic era, mask-wearing becomes a matter of personal choice. Mallinas et al. [65] found that empathy, trust in healthcare professionals, and perceived normativity of mask-wearing were associated with pro-mask attitudes, as well as demographic variables. Lee [66] suggested that a code of conduct and risk communication strategies for COVID-19 in Korea should be developed according to the stage of the crisis alert and customized for each social group, and a consistent, unified, and scientific evidence-based message with real-life applicability should be systematically developed, monitored, and evaluated. Along with professional and governmental efforts, the general public must be consistent and flexible without being overly sensitive [67].

### 4.4. Demographic Factors of Mask-Wearing Behaviors

In this study, the daily choice of face mask was affected mainly by age and occupation. However, discomfort due to masks, anxiety when not wearing a mask, and future mask-wearing plans were affected by occupation and gender. Although occupation in this study was highly correlated with age, as listed in Table 5, the results showed that age and occupation affected different aspects of mask-wearing behaviors.

Age and gender are the two main demographic factors affecting mask-wearing attitudes and behaviors. However, as each study has its own research design and methodology, the results cannot be directly compared. Age affected simple daily choice in this study, which is consistent with Asri et al. [38] who found that age was the only demographic measure related to significant differences in mask-wearing on/off in their large survey of hospital employees in Switzerland. Other factors, such as gender, education, and occupation, showed relatively minor differences. However, age did not mainly affect the perception level of mask-wearing behavior in this study, although speech discomfort was affected by age (*p* = 0.032). This is not consistent with the results that older people tended to follow socially accepted behavior in their area of residence in Spain [68], and that older adults reported greater mask-related worrying [60]. Contrary to the results of this study, Han et al. [13] reported that age and gender did not affect the explicit attitude toward wearing a mask with statistical significance. While for men protecting others plays a significant role, for women, self-protection is more important [64]. Women had better preventive behaviors wearing a face mask than men in Iran [69]. In the US, gender, age, and location factored into whether shoppers wore a mask voluntarily [70,71], and mask-wearing patterns during the COVID-19 pandemic were differently shaped by racial and ethnic background and gender [72]. Mallinas et al. [65] found that demographic variables such as political conservatism, younger age, and gender predicted anti-mask attitudes but were unrelated to pro-mask attitudes. 

### 4.5. Limitations and Future Studies

This study had several limitations. First, the appropriate distribution of people by gender, age, and occupation was not performed during the sampling process. Therefore, these results should be cautiously interpreted.

Second, a more detailed categorization of demographic factors could be recommended for improving the face mask itself and the usability of mask-wearing. Respondents’ health status, specifically their experience of COVID-19, should be considered in future studies. Variables related to general socioeconomic status were excluded because wearing masks was compulsory in indoor and outdoor public places nationwide at the time of the research survey. However, in future studies, it will be necessary to include and examine these variables more closely. 

Third, the data collected in this study cannot be considered representative of mask-wearing behavior in Seoul and Gwangju. This study was distributed nationwide in the form of an online questionnaire; however, no regional categories were provided for further classification. This was due to the nationwide mask-wearing policy; however, regional effects on mask-wearing behavior may exist because of the regional spread of COVID-19 or the local environment. Therefore, it is necessary to analyze and interpret the regional aspects in future research.

Fourth, this study was a survey to capture actual mask-wearing behavior from the perspective of end users. Therefore, based on the results of this study, it is expected that studies on improvement measures and ease of wearing a mask according to the prolonged use of a mask will proceed.

## 5. Conclusions

In Korea, wearing masks in public places has become the norm during the prolonged COVID-19 pandemic. This cross-sectional study investigated the mask-wearing behavior of Koreans after wearing a mask in public spaces for two years due to COVID-19.

The respondents selected their face masks based on season, gender, age, occupation, mask-wearing hours, mask filter performance, mask shape, and mask color. The general discomfort caused by wearing a mask was divided into physical and speech discomfort, and it was not correlated with anxiety when not wearing a face mask. Speech discomfort caused by wearing a mask was correlated with general discomfort, clear speech, vocal pain, anxiety, and indoor mask-off plans. Anxiety when not wearing a mask appeared to affect both indoor and outdoor mask-off plans. The more uncomfortable and less anxious respondents were when not wearing a mask, the sooner they wanted to discontinue wearing masks indoors and outdoors.

It is expected that the use of masks will continue in the future, and that there may be differences in the place and time of the use of masks in Korea and around the world due to new infectious diseases and fine dust. Facial masks can be worn more comfortably and conveniently if the discomfort and anxiety of wearing a mask are worked on in the future considering various behaviors when wearing a mask. In addition to a detailed survey of the actual situation, measures to improve communication difficulties when wearing masks should also be investigated. It is necessary to examine in detail the change in the perception of mask-wearing behavior at other times when the epidemic situation has worsened or weakened.

## Figures and Tables

**Table 1:** Descriptive statistics of survey respondents (*n* = 433).

Questions	Category	*n*	%
Q1. Gender	Male	153	35.3
	Female	280	64.7
Q2. Age groups	18–39	206	47.6
	40–59	143	33.0
	≥60	84	19.4
Q3. Occupation	Student	181	41.8
	Employed	161	37.2
	Homemaker	62	14.3
	Freelancer, Farmer, etc.	29	6.7

**Table 2:** Survey results: daily choice of face mask.

Questions	Variables				*n*	%	χ^2^ (*p*)
Q4. Mask-wearing hours in a day (Duration)	<3 h				81	18.7	116.319 (*p* < 0.0005)
	3–6 h				118	27.3	
	6–9 h				137	31.6	
	9–12 h				91	21.0	
	None of the above				6	1.4	
Q5. Mask filter performance	KF-AD/Surgical				37	8.5	1072.162 (*p* < 0.0005)
	KF-80				27	6.2	
	KF-94				358	82.7	
	Fabric				9	2.1	
	None of the above				2	0.5	
Q6. Mask shape	Vertical folding (VF)				168	38.8	41.159 (*p* < 0.0005)
	Horizontal folding (HF)				183	42.3	
	No preference				82	18.9	
Q7. Mask color	White				317	73.2	559.656 (*p* < 0.0005)
	Black				79	18.2	
	Gray scale				23	5.3	
	Chromatic color				14	3.2	
Q8. Seasonal mask choice		Summer	χ^2^(*p*)	Winter	χ^2^(*p*)	Spring/Autumn	χ^2^(*p*)
	KF-AD VF	57 (13.2%)	84.661 (*p* < 0.0005)	42 (9.7%)	249.169 (*p* < 0.0005)	43 (9.9%)	161.515 (*p* < 0.0005)
	KF-AD HF	73 (16.9%)		61 (14.1%)		55 (12.7%)	
	Surgical	102 (23.6%)		25 (5.8%)		44 (10.2%)	
	KF-80 VF	31 (7.2%)		31 (7.2%)		38 (8.8%)	
	KF-80 HF	17 (3.9%)		11 (2.5%)		16 (3.7%)	
	KF-94 VF	70 (16.2%)		117 (27.0%)		109 (25.2%)	
	KF-94 HF	83 (19.2%)		146 (33.7%)		128 (29.6%)	

**Table 3:** Survey results: discomfort due to a face mask.

Questions	Variables	*n*	%	χ^2^ (*p*)
Q9. Discomfort when wearing a mask in general	Disagree	14	3.2	128.143 (*p* < 0.0005)
	Slightly agree	53	12.2	
	Moderately agree	110	25.4	
	Agree	147	33.9	
	Strongly agree	109	25.2	
Q10. Select all the discomforts when wearing a mask	No discomfort	23	2.7	
	Shortness of breath	216	25.5	
	Difficulty speaking	188	22.2	
	Mask movement when speaking	159	18.8	
	Ear pain due to mask string	193	22.8	
	None of the above	69	8.1	
	Total	853	100.0	
Q11. Discomfort when talking while wearing a mask	Disagree	16	3.7	148.513 (*p* < 0.0005)
	Slightly agree	47	10.9	
	Moderately agree	101	23.3	
	Agree	163	37.6	
	Strongly agree	106	24.5	
Q12. Select all the discomforts when talking while wearing a mask	Cannot hear well	247	36.5	
	Speak repeatedly	189	28.0	
	Speak loudly	209	30.9	
	None of the above	31	4.6	
	Total	676	100.0	
Q13. When speaking while wearing a mask, trying to pronounce clearly	Disagree	28	6.5	108.351 (*p* < 0.0005)
	Slightly agree	54	12.5	
	Moderately agree	116	26.8	
	Agree	150	34.6	
	Strongly agree	85	19.6	
Q14. Vocal pain when speaking while wearing a mask	Disagree	114	26.3	32.000 (*p* < 0.0005)
	Slightly agree	100	23.1	
	Moderately agree	91	21.0	
	Agree	84	19.4	
	Strongly agree	44	10.2	

**Table 4:** Survey results: anxiety due to not wearing a mask and plan to wear a mask.

Questions	Variables	*n*	%	χ^2^ (*p*)
Q15. Anxiety when not wearing a mask	Disagree	74	17.1	24.910 (*p* < 0.0005)
	Slightly agree	57	13.2	
	Moderately agree	88	20.3	
	Agree	119	27.5	
	Strongly agree	95	21.9	
Q16. Select all the reasons for your anxiety when not wearing a mask	Increased risk of droplet transmission	316	50.2	
	Negative public opinion on not wearing a mask	185	29.4	
	Psychological anxiety due to wearing habits for 2 years	118	18.8	
	None of the above	10	1.6	
	Total	629	100.0	
Q17. What are the plans for wearing a mask “indoors” after the expert group declares the end of COVID-19?	Discontinue wearing immediately	119	27.5	115.949 (*p* < 0.0005)
	Discontinue within 3 months	152	35.1	
	Discontinue within 6 months	76	17.6	
	Discontinue within 12 months	23	5.3	
	Continue to wear it for more than 1 year	63	14.5	
Q18. What are the plans for wearing a mask “outdoors” after the expert group declares the end of COVID-19?	Discontinue wearing immediately	139	32.1	133.986 (*p* < 0.0005)
	Discontinue within 3 months	148	34.2	
	Discontinue within 6 months	60	13.9	
	Discontinue within 12 months	26	6.0	
	Continue to wear it for more than 1 year	60	13.9	

**Table 5:** Spearman’s rank correlation coefficients (*n* = 433, ** *p* < 0.01, * *p* < 0.05).

Questions		Q1	Q2	Q3	Q4	Q5	Q6	Q7	Q8 S	Q8 W	Q8 A	Q9	Q11	Q13	Q14	Q15	Q17	Q18
Q1. Gender	ρ	1.000	−0.085	−0.014	0.050	0.082	−0.074	−0.045	0.017	0.182 **	0.126 **	0.013	0.097 *	0.112 *	0.225 **	0.252 **	0.266 **	0.219 **
	*p*	.	0.079	0.775	0.296	0.089	0.122	0.351	0.721	0.000	0.009	0.788	0.043	0.020	0.000	0.000	0.000	0.000
Q2. Age	ρ		1.000	0.826 **	−0.400 **	0.079	0.032	−0.023	−0.178 **	−0.218 **	−0.190 **	−0.075	−0.123 *	−0.029	0.027	0.039	0.011	−0.039
	*p*		.	0.000	0.000	0.101	0.503	0.639	0.000	0.000	0.000	0.118	0.010	0.552	0.572	0.415	0.822	0.421
Q3. Occupation	ρ			1.000	−0.407 **	0.003	0.040	0.003	−0.144 **	−0.171 **	−0.146 **	−0.051	−0.126 **	−0.011	0.068	0.092	0.036	−0.042
	*p*			.	0.000	0.946	0.409	0.956	0.003	0.000	0.002	0.285	0.009	0.817	0.156	0.055	0.461	0.384
Q4. Hours	ρ				1.000	−0.034	0.029	−0.013	0.165 **	0.168 **	0.148 **	0.034	0.059	0.044	0.022	−0.053	−0.005	0.023
	*p*				.	0.477	0.548	0.788	0.001	0.000	0.002	0.483	0.220	0.359	0.642	0.269	0.919	0.629
Q5. Filter	ρ					1.000	−0.091	−0.027	0.226 **	0.253 **	0.278 **	−0.042	−0.040	0.053	0.004	−0.006	0.076	0.022
	*p*					.	0.058	0.573	0.000	0.000	0.000	0.388	0.409	0.269	0.935	0.908	0.112	0.644
Q6. Shape	ρ						1.000	−0.178 **	0.119 *	0.168 **	0.142 **	0.013	−0.012	−0.093	−0.041	−0.084	−0.049	−0.019
	*p*						.	0.000	0.014	0.000	0.003	0.786	0.808	0.052	0.399	0.082	0.313	0.689
Q7. Color	ρ							1.000	−0.105 *	−0.155 **	−0.160 **	0.029	0.013	0.028	0.033	−0.057	−0.081	−0.128 **
	*p*							.	0.029	0.001	0.001	0.543	0.780	0.560	0.493	0.233	0.094	0.007
Q8. Summer	ρ								1.000	0.635 **	0.710 **	−0.064	−0.048	−0.004	−0.010	0.071	0.134 **	0.164 **
	*p*								.	0.000	0.000	0.182	0.315	0.937	0.839	0.139	0.005	0.001
Q8. Winter	ρ									1.000	0.894 **	−0.035	0.021	0.025	0.081	0.072	0.068	0.038
	*p*									.	0.000	0.463	0.664	0.603	0.091	0.135	0.155	0.425
Q8. Spring/ Autumn	ρ										1.000	−0.026	−0.014	0.018	0.042	0.041	0.094	0.060
	*p*										.	0.594	0.775	0.712	0.388	0.399	0.050	0.210
Q9. General Discomfort	ρ											1.000	0.580 **	0.274 **	0.250 **	0.056	−0.168 **	−0.194 **
	*p*											.	0.000	0.000	0.000	0.244	0.000	0.000
Q11 Speech Discomfort	ρ												1.000	0.349 **	0.330 **	0.100 *	−0.105 *	−0.086
	*p*												.	0.000	0.000	0.038	0.029	0.074
Q13. Clear Speech	ρ													1.000	0.403 **	0.208 **	0.016	−0.008
	*p*													.	0.000	0.000	0.748	0.872
Q14. Vocal Pain	ρ														1.000	0.246 **	0.027	−0.030
	*p*														.	0.000	0.570	0.527
Q15. Anxiety	ρ															1.000	0.292 **	0.291 **
	*p*															.	0.000	0.000
Q17. Mask-off: Indoor	ρ																1.000	0.777 **
	*p*																.	0.000
Q18. Mask-off: Outdoor	ρ																	1.000
	*p*																	.

**Table 6:** Chi-squared test results according to age.

Questions	Variables	20’s–30’s	40’s–50’s	60’s–80’s	Total	χ^2^ (*p*)
Q4. Duration	<3 h	9 (4.4%)	25 (17.5%)	47 (56.0%)	81 (18.7%)	123.508 (*p* < 0.0005)
	3–6 h	59 (28.6%)	32 (22.4%)	27 (32.1%)	118 (27.3%)	
	6–9 h	80 (38.8%)	51 (35.7%)	6 (7.1%)	137 (31.6%)	
	9–12 h	54 (26.2%)	34 (23.8%)	3 (3.6%)	91 (21.0%)	
	None of the above	4 (1.9%)	1 (0.7%)	1 (1.2%)	6 (1.4%)	
	Total	206 (100%)	143(100%)	84 (100%)	433 (100%)	
Q7. Color	White	149 (72.3%)	100 (69.9%)	68 (81.0%)	317 (73.2%)	22.653 (*p* = 0.001)
	Black	46 (22.3%)	30 (21.0%)	3 (3.6%)	79 (18.2%)	
	Gray scale	5 (2.4%)	10 (7.0%)	8 (9.5%)	23 (5.3%)	
	Chromatic color	6 (2.9%)	3 (2.1%)	5 (6.0%)	14 (3.2%)	
	Total	206 (100%)	143 (100%)	84 (100%)	433 (100%)	
Q8. Summer	KF-AD VF	23 (11.2%)	14 (9.8%)	20 (23.8%)	57 (13.2%)	43.621 (*p* < 0.0005)
	KF-AD HF	27 (13.1%)	28 (19.6%)	18 (21.4%)	73 (16.9%)	
	Surgical	54 (26.2%)	27 (18.9%)	21 (25.0%)	102 (23.6%)	
	KF-80 VF	10 (4.9%)	9 (6.3%)	12 (14.3%)	31 (7.2%)	
	KF-80 HF	5 (2.4%)	8 (5.6%)	4 (4.8%)	17 (3.9%)	
	KF-94 VF	42 (20.4%)	23 (16.1%)	5 (6.0%)	70 (16.2%)	
	KF-94 HF	45 (21.8%)	34 (23.8%)	4 (4.8%)	83 (19.2%)	
	Total	206 (100%)	143 (100%)	84 (100%)	433 (100%)	
Q8. Winter	KF-AD VF	17 (8.3%)	5 (3.5%)	20 (23.8%)	42 (9.7%)	80.779 (*p* < 0.0005)
	KF-AD HF	19 (9.2%)	15 (10.5%)	27 (32.1%)	61 (14.1%)	
	Surgical	15 (7.3%)	4 (2.8%)	6 (7.1%)	25 (5.8%)	
	KF-80 VF	11 (5.3%)	11 (7.7%)	9 (10.7%)	31 (7.2%)	
	KF-80 HF	7 (3.4%)	2 (1.4%)	2 (2.4%)	11 (2.5%)	
	KF-94 VF	64 (31.1%)	42 (29.4%)	11 (13.1%)	117 (27.0%)	
	KF-94 HF	73 (35.4%)	64 (44.8%)	9 (10.7%)	146 (33.7%)	
	Total	206 (100%)	143 (100%)	84 (100%)	433 (100%)	
Q8. Spring/Autumn	KF-AD VF	17 (8.3%)	6 (4.2%)	20 (23.8%)	43 (9.9%)	65.267 (*p* < 0.0005)
	KF-AD HF	19 (9.2%)	15 (10.5%)	21 (25.0%)	55 (12.7%)	
	Surgical	25 (12.1%)	7 (4.9%)	12 (14.3%)	44 (10.2%)	
	KF-80 VF	14 (6.8%)	15 (10.5%)	9 (10.7%)	38 (8.8%)	
	KF-80 HF	7 (3.4%)	6 (4.2%)	3 (3.6%)	16 (3.7%)	
	KF-94 VF	62 (30.1%)	37 (25.9%)	10 (11.9%)	109 (25.2%)	
	KF-94 HF	62 (30.1%)	57 (39.9%)	9 (10.7%)	128 (29.6%)	
	Total	206 (100%)	143 (100%)	84 (100%)	433 (100%)	
Q11. Speech Discomfort	Disagree	7 (3.4%)	5 (3.5%)	4 (4.8%)	16 (3.7%)	16.784 (*p* = 0.032)
	Slightly agree	14 (6.8%)	21 (14.7%)	12 (14.3%)	47 (10.9%)	
	Moderately agree	53 (25.7%)	28 (19.6%)	20 (23.8%)	101 (23.3%)	
	Agree	68 (33.0%)	59 (41.3%)	35 (41.7%)	162 (37.4%)	
	Strongly agree	64 (31.1%)	30 (21.0%)	13 (15.5%)	107 (24.7%)	
	Total	206 (100%)	143 (100%)	84 (100%)	433 (100%)	

**Table 7:** Chi-squared test results according to occupation.

Questions	Variables	Student	Employed	Homemaker	Freelancer	Total	χ^2^ (*p*)
Q4. Duration	<3 h	4 (2.2%)	19 (11.8%)	42 (67.7%)	16 (55.2%)	81 (18.7%)	176.492 (*p* < 0.0005)
	3–6 h	56 (30.9%)	39 (24.2%)	16 (25.8%)	7 (24.1%)	118 (27.3%)	
	6–9 h	75 (41.4%)	55 (34.2%)	3 (4.8%)	4 (13.8%)	137 (31.6%)	
	9–12 h	43 (23.8%)	46 (28.6%)	0 (0%)	2 (6.9%)	91 (21.0%)	
	None of the above	3 (1.7%)	2 (1.2%)	1 (1.6%)	0 (0%)	6 (1.4%)	
	Total	181 (100)	161 (100%)	62 (100%)	29 (100%)	433 (100%)	
Q7. Color	White	134 (74.0%)	112 (69.6%)	45 (72.6%)	26 (89.7%)	317 (73.2%)	16.910 (*p* = 0.050)
	Black	40 (22.1%)	30 (18.6%)	8 (12.9%)	1 (3.4%)	79 (18.2%)	
	Gray scale	4 (2.2%)	12 (7.5%)	6 (9.7%)	1 (3.4%)	23 (5.3%)	
	Chromatic color	3 (1.7%)	7 (4.3%)	3 (4.8%)	1 (3.4%)	14 (3.2%)	
	Total	181 (100%)	161 (100%)	62 (100%)	29 (100%)	433 (100%)	
Q8. Summer	KF-AD VF	19 (10.5%)	20 (12.4%)	13 (21.0%)	5 (17.2%)	57 (13.2%)	35.738 (*p* = 0.008)
	KF-AD HF	25 (13.8%)	30 (18.6%)	14 (22.6%)	4 (13.8%)	73 (16.9%)	
	Surgical	49 (27.1%)	29 (18.0%)	14 (22.6%)	10 (34.5%)	102 (23.6%)	
	KF-80 VF	7 (3.9%)	14 (8.7%)	8 (12.9%)	2 (6.9%)	31 (7.2%)	
	KF-80 HF	4 (2.2%)	7 (4.3%)	3 (4.8%)	3 (10.3%)	17 (3.9%)	
	KF-94 VF	38 (21.0%)	26 (16.1%)	3 (4.8%)	3 (10.3%)	70 (16.2%)	
	KF-94 HF	39 (21.5%)	35 (21.7%)	7 (11.3%)	2 (6.9%)	83 (19.2%)	
	Total	181 (100%)	161 (100%)	62 (100%)	29 (100%)	433 (100%)	
Q8. Winter	KF-AD VF	16 (8.8%)	11 (6.8%)	8 (12.9%)	7 (24.1%)	42 (9.7%)	35.392 (*p* = 0.008)
	KF-AD HF	17 (9.4%)	22 (13.7%)	16 (25.8%)	6 (20.7%)	61 (14.1%)	
	Surgical	12 (6.6%)	8 (5.0%)	3 (4.8%)	2 (6.9%)	25 (5.8%)	
	KF-80 VF	9 (5.0%)	13 (8.1%)	7 (11.3%)	2 (6.9%)	31 (7.2%)	
	KF-80 HF	6 (3.3%)	1 (0.6%)	3 (4.8%)	1 (3.4%)	11 (2.5%)	
	KF-94 VF	54 (29.8%)	44 (27.3%)	13 (21.0%)	6 (20.7%)	117 (27.0%)	
	KF-94 HF	67 (37.0%)	62 (38.5%)	12 (19.4%)	5 (17.2%)	146 (33.7%)	
	Total	181 (100%)	161 (100%)	62 (100%)	29 (100%)	433 (100%)	
Q8. Spring/ Autumn	KF-AD VF	16 (8.8%)	15 (9.3%)	8 (12.9%)	4 (13.8%)	43 (9.9%)	41.779 (*p* = 0.001)
	KF-AD HF	16 (8.8%)	19 (11.8%)	13 (21.0%)	7 (24.1%)	55 (12.7%)	
	Surgical	22 (12.2%)	12 (7.5%)	5 (8.1%)	5 (17.2%)	44 (10.2%)	
	KF-80 VF	10 (5.5%)	15 (9.3%)	12 (19.4%)	1 (3.4%)	38 (8.8%)	
	KF-80 HF	5 (2.8%)	5 (3.1%)	5 (8.1%)	1 (3.4%)	16 (3.7%)	
	KF-94 VF	55 (30.4%)	42 (26.1%)	5 (8.1%)	7 (24.1%)	109 (25.2%)	
	KF-94 HF	57 (31.5%)	53 (32.9%)	14 (22.6%)	4 (13.8%)	128 (29.6%)	
	Total	181 (100%)	161 (100%)	62 (100%)	29 (100%)	433 (100%)	
Q11. Speech Discomfort	Disagree	8 (4.4%)	4 (2.5%)	1 (1.6%)	3 (10.3%)	16 (3.7%)	23.135 (*p* = 0.027)
	Slightly agree	13 (7.2%)	22 (13.7%)	6 (9.7%)	6 (20.7%)	47 (10.9%)	
	Moderately agree	42 (23.2%)	36 (22.4%)	18 (29.0%)	5 (17.2%)	101 (23.3%)	
	Agree	60 (33.1%)	65 (40.4%)	24 (38.7%)	13 (44.8%)	162 (37.4%)	
	Strongly agree	58 (32.0%)	34 (21.1%)	13 (21.0%)	2 (6.9%)	107 (24.7%)	
	Total	181 (100%)	143 (100%)	62 (100%)	29 (100%)	433 (100%)	
Q14. Vocal Pain	Disagree	56 (30.9%)	29 (18.0%)	14 (22.6%)	15 (51.7%)	114 (13.8%)	33.068 (*p* = 0.001)
	Slightly agree	46 (25.4%)	38 (23.6%)	12 (19.4%)	4 (13.8%)	100 (23.1%)	
	Moderately agree	41 (22.7%)	28 (17.4%)	16 (25.8%)	6 (20.7%)	91 (21.0%)	
	Agree	22 (12.2%)	44 (27.3%)	14 (22.6%)	4 (13.8%)	84 (19.4%)	
	Strongly agree	16 (8.8%)	22 (13.7%)	6 (9.7%)	0 (0%)	44 (10.2%)	
	Total	181 (100%)	161 (100%)	62 (100%)	29 (100%)	433 (100%)	
Q15. Anxiety	Disagree	43 (23.8%)	17 (10.6%)	7 (11.3%)	7 (24.1%)	74 (17.1%)	31.466 (*p* = 0.002)
	Slightly agree	18 (9.9%)	26 (16.1%)	9 (14.5%)	4 (13.8%)	57 (13.2%)	
	Moderately agree	38 (21.0%)	36 (22.4%)	5 (8.1%)	9 (31.0%)	88 (20.3%)	
	Agree	48 (26.5%)	49 (30.4%)	17 (27.4%)	5 (17.2%)	119 (27.5%)	
	Strongly agree	34 (18.8%)	33 (20.5%)	24 (38.7%)	4 (13.8%)	95 (21.9%)	
	Total	181 (100%)	161 (100%)	62 (100%)	29 (100%)	433 (100%)	
Q17. Mask-off Plans: Indoor	Discontinue wearing immediately	52 (28.7%)	47 (29.2%)	8 (12.9%)	12 (41.4%)	119 (27.5%)	25.873 (*p* = 0.011)
	Discontinue within 3 months	61 (33.7%)	60 (37.3%)	22 (35.5%)	9 (31.0%)	152 (35.1%)	
	Discontinue within 6 months	30 (16.6%)	28 (17.4%)	13 (21.0%)	5 (17.2%)	76 (17.6%)	
	Discontinue within 12 months	17 (9.4%)	2 (1.2%)	4 (6.5%)	0 (0%)	23 (5.3%)	
	Continue to wear it for more than 1 yr	21 (11.6%)	24 (14.9%)	15 (24.2%)	3 (10.3%)	63 (14.5%)	
	Total	181 (100%)	161 (100%)	62 (100%)	29 (100%)	433 (100%)	

**Table 8:** Chi-squared test results according to gender.

Questions	Variables	Men	Women	Total	χ^2^ (*p*)
Q5. Filter performance	KF-AD	18 (11.8)	19 (6.8)	37 (8.5)	17.799 (*p* = 0.001)
	KF-80	15 (9.8)	12 (4.3)	27 (6.2)	
	KF-94	112 (73.2)	246 (87.9)	358 (82.7)	
	Fabric	6 (3.9)	3 (1.1)	9 (2.1)	
	None of the above	2 (1.3)	0 (0%)	2 (0.5)	
	Total	153 (100)	280 (100)	433 (100)	
Q8. Winter	KF-AD VF	20 (13.1)	22 (7.9)	42 (9.7)	22.907 (*p* = 0.001)
	KF-AD HF	31 (20.3)	30 (10.7)	61 (14.1)	
	Surgical	15 (9.8)	10 (3.6)	25 (5.8)	
	KF-80 VF	10 (6.5)	21 (7.5)	31 (7.2)	
	KF-80 HF	2 (1.3)	9 (3.2)	11 (2.5)	
	KF-94 VF	35 (22.9)	82 (29.3)	117 (27.0)	
	KF-94 HF	40 (26.1)	106 (37.9)	146 (33.7)	
	Total	153 (100)	280 (100)	433 (100)	
Q8. Spring/Autumn	KF-AD VF	18 (11.8)	25 (8.9)	43 (9.9)	18.771 (*p* = 0.005)
	KF-AD HF	29 (19.0)	26 (9.3)	55 (12.7)	
	Surgical	21 (13.7)	23 (8.2)	44 (10.2)	
	KF-80 VF	9 (5.9)	29 (10.4)	38 (8.8)	
	KF-80 HF	2 (1.3)	14 (5.0)	16 (3.7)	
	KF-94 VF	35 (22.9)	74 (26.4)	109 (25.2)	
	KF-94 HF	39 (25.5)	89 (31.8)	128 (29.6)	
	Total	153 (100)	280 (100)	433 (100)	
Q11. Speech Discomfort	Disagree	11 (7.2)	5 (1.8)	16 (3.7)	15.118 (*p* = 0.004)
	Slightly agree	24 (15.7)	23 (8.2)	47 (10.9)	
	Moderately agree	30 (19.6)	71 (25.4)	101 (23.3)	
	Agree	53 (34.6)	109 (38.9)	162 (37.4)	
	Strongly agree	35 (22.9)	72 (25.7)	107 (24.7)	
	Total	153 (100)	280 (100)	433 (100)	
Q14. Vocal Pain	Disagree	54 (35.3)	60 (21.4)	114 (26.3)	23.215 (*p* < 0.0005)
	Slightly agree	42 (27.5)	58 (20.7)	100 (23.1)	
	Moderately agree	32 (20.9)	59 (21.1)	91 (21.0)	
	Agree	17 (11.1)	67 (23.9)	84 (19.4)	
	Strongly agree	8 (5.2)	36 (12.9)	44 (10.2)	
	Total	153 (100)	280 (100)	433 (100)	
Q15. Anxiety	Disagree	42 (27.5)	32 (11.4)	74 (17.1)	31.517 (*p* < 0.0005)
	Slightly agree	21 (13.7)	36 (12.9)	57 (13.2)	
	Moderately agree	39 (25.5)	49 (17.5)	88 (20.3)	
	Agree	32 (20.9)	87 (31.1)	119 (27.5)	
	Strongly agree	19 (12.4)	76 (27.1)	95 (21.9)	
	Total	153 (100)	280 (100)	433 (100)	
Q17. Mask-off Plans: Indoor	Discontinue wearing immediately	58 (37.9)	61 (21.8)	119 (27.5)	36.258 (*p* < 0.0005)
	Discontinue within 3 months	66 (43.1)	86 (30.7)	152 (35.1)	
	Discontinue within 6 months	15 (9.8)	61 (21.8)	76 (17.6)	
	Discontinue within 12 months	2 (1.3)	21 (7.5)	23 (5.3)	
	Continue to wear it for more than 1 yr	12 (7.8)	51 (18.2)	63 (14.5)	
	Total	153 (100)	280 (100)	433 (100)	
Q18. Mask-off Plans: Outdoor	Discontinue wearing immediately	63 (41.2)	76 (27.1)	139 (32.1)	24.077 (*p* < 0.0005)
	Discontinue within 3 months	61 (39.9)	87 (31.1)	148 (34.2)	
	Discontinue within 6 months	13 (8.5)	47 (16.8)	60 (13.9)	
	Discontinue within 12 months	6 (3.9)	20 (7.1)	26 (6.0)	
	Continue to wear it for more than 1 yr	10 (6.5)	50 (17.9)	60 (13.9)	
	Total	153 (100)	280 (100)	433 (100)	

**Table 9:** Other discomforts and anxiety (subjective responses).

Questions	Subjective Responses	*n*
Q10. The discomforts when wearing a mask: None of the above (Total 69)	Foggy glasses	28
	Sweat and moisture	11
	Skin disorder	7
	Makeup smudging	5
	Facial discomfort, Discomfort under the chin, Nose discomfort, Facial tightness	7
	Sleepier when wearing a mask/When the weather is cool, it’s okay; when it’s hot, it’s hard/Inconvenient and uncomfortable due to insufficient oxygen supply/Contamination after eating is a concern/Smelly/Hot	6
Q12. The discomforts when talking while wearing a mask: None of the above (Total 31)	Moisture in the mask	4
	Mouth shape and facial expression are not visible	6
	A mask moves down	5
	Short of breath	4
	Smells	2
	Discomfort when talking/Ear pain/ Foggy glasses/Mouth touching the mask during conversation/No discomfort/ Difficulty hearing and pronunciation is not correct	3
	No discomfort	3
	I do not know	1
Q16. The reasons for your anxiety when not wearing a mask: None of the above (Total 10)	Due to the nature of the job, it must be worn at all times	1
	I wear it for fear that the other person would be uncomfortable	1
	None	8

## Data Availability

Not applicable.

## References

[1] WHO WHO Director-General’s Opening Remarks at the Media Briefing on COVID-19—11 March 2020. https://www.who.int/director-general/speeches/detail/who-director-general-s-opening-remarks-at-the-media-briefing-on-covid-19.

[2] (2020). KDCA, Masks Are a Must Now! I Use it with Prime Minister Chung!.

[3] MOHW Ministry of Health and Wellbeing, Press Reference. http://ncov.mohw.go.kr/upload/viewer/skin/doc.html?fn=1651197423233_20220429105703.pdf&rs=/upload/viewer/result/202205/.

[4] Kim S., Kim Y.-J., Peck K.R., Ko Y., Lee J., Jung E. (2020). Keeping low reproductive number despite the rebound population mobility in Korea, a country never under lockdown during the COVID-19 pandemic. Int. J. Environ. Res. Public Health.

[5] Sachs J., Schmidt-Traub G., Kroll C., Lafortune G., Fuller G., Woelm F. (2021). Sustainable Development Report 2020: The Sustainable Development Goals and COVID-19 Includes the SDG Index and Dashboards.

[6] Lim B., Kyoungseo Hong E., Mou J., Cheong I. (2021). COVID-19 in Korea: Success based on past failure. Asian Econ. Pap..

[7] Kang Y.-J. (2021). COVID-19 in South Korea: Proper Timing for Easing Mask Mandates After COVID-19 Vaccination. Disaster Med. Public Health Prep..

[8] Kang S., Guak S., Bataa A., Kim D., Jung Y., Shin J., Lee K. (2020). Mask-wearing characteristics an COVID-19 in indoor and outdoor environments in Seoul in 2020. J. Environ. Health Sci..

[9] Chang H.J., Min S., Woo H., Yurchisin J. (2021). Mask-Wearing Behavior During the COVID-19 Pandemic: A Cross-Cultural Comparison Between the United States and South Korea. Fam. Consum. Sci. Res. J..

[10] Chung J.-B., Kim B.-J., Kim E.-S. (2021). Mask Wearing Behavior and COVID-19: Synthetic Effects of Individualism and Collectivism in Korea. Res. Sq..

[11] Mo Y., Park H.S. (2021). COVID-19 and Public Masking Compliance in Korea: We-ness and Individualism-Collectivism at the Individual Level. Health Commun..

[12] Kim H.J., Han S.Y. (2022). Mask Use during the COVID-19 Pandemic: A Descriptive Survey in South Korea. Nurs. Health Sci..

[13] Han E.J., Na J., Bang J., Sul S. (2022). Explicit and implicit attitudes towards mask wearing in the midst of COVID-19. Korean J. Soc. Personal. Psychol..

[14] Ahmad M.F., Wahab S., Ahmad F.A., Alam M.I., Ather H., Siddiqua A., Ashraf S.A., Shaphe M.A., Khan M.I., Beg R.A. (2021). A novel perspective approach to explore pros and cons of face mask in prevention the spread of SARS-CoV-2 and other pathogens. Saudi Pharm. J..

[15] O’Connor Z., Huellewig D., Sithiyopasakul P., Morris J.A., Gan C., Ballard D.H. (2020). 3D printed mask extenders as a supplement to isolation masks to relieve posterior auricular discomfort: An innovative 3D printing response to the COVID-19 pandemic. 3D Print. Med..

[16] Das A., Kumar S., Sil A., Jafferany M. (2020). Skin changes attributed to protective measures against COVID-19: A compilation. Dermatol. Ther..

[17] Park M., Kim H., Kim S., Lee J., Kim S., Byun J.W., Hwang-Bo J., Park K.H. (2021). Changes in skin wrinkles and pores due to long-term mask wear. Ski. Res. Technol..

[18] Magee M., Lewis C., Noffs G., Reece H., Chan J.C., Zaga C.J., Paynter C., Birchall O., Rojas Azocar S., Ediriweera A. (2020). Effects of face masks on acoustic analysis and speech perception: Implications for peri-pandemic protocols. J. Acoust. Soc. Am..

[19] Jeong J., Kim M., Kim Y. (2020). Changes on Speech Transmission Characteristics by Types of Mask. Audiol. Speech Res..

[20] Yi H., Pingsterhaus A., Song W. (2021). Effects of wearing face masks while using different speaking styles in noise on speech intelligibility during the COVID-19 pandemic. Front. Psychol..

[21] Caniato M., Marzi A., Gasparella A. (2021). How much COVID-19 face protections influence speech intelligibility in classrooms?. Appl. Acoust..

[22] Chmelík V., Urbán D., Zelem L., Rychtáriková M. (2021). Effect of Mouth Mask and Face Shield on Speech Spectrum in Slovak Language. Appl. Sci..

[23] Nguyen D.D., McCabe P., Thomas D., Purcell A., Doble M., Novakovic D., Chacon A., Madill C. (2021). Acoustic voice characteristics with and without wearing a facemask. Sci. Rep..

[24] Knowles T., Badh G. (2022). The impact of face masks on spectral acoustics of speech: Effect of clear and loud speech styles. J. Acoust. Soc. Am..

[25] Corey R.M., Jones U., Singer A.C. (2021). Comparison of the acoustic effects of face masks on speech. Hear. J..

[26] Brown V.A., Van Engen K.J., Peelle J.E. (2021). Face mask type affects audiovisual speech intelligibility and subjective listening effort in young and older adults. Cogn. Res. Princ. Implic..

[27] Yi H., Pingsterhaus A., Song W. (2021). The adverse effect of wearing a face mask during the COVID-19 pandemic and benefits of wearing transparent face masks and using clear speech on speech intelligibility. PsyArXiv.

[28] Thibodeau L.M., Thibodeau-Nielsen R.B., Tran C.M.Q., de Souza Jacob R.T. (2021). Communicating during COVID-19: The effect of transparent masks for speech recognition in noise. Ear Hear..

[29] Atcherson S.R., McDowell B.R., Howard M.P. (2021). Acoustic effects of non-transparent and transparent face coverings. J. Acoust. Soc. Am..

[30] Tsantani M., Podgajecka V., Gray K.L., Cook R. (2022). How does the presence of a surgical face mask impair the perceived intensity of facial emotions?. PLoS ONE.

[31] Ma L., Kim M.-S. (2020). A Study of The Purchasing Tendency of Healthcare Masks Based on The User-centered Design Concept-centered on the Form and Color of the Mask. J. Korea Converg. Soc..

[32] Liu S.-F., Chang J.-F., Wang M.-H. (2021). Mask Design for Life in the Midst of COVID-19. Sustainability.

[33] Ipaki B., Merrikhpour Z., Taheri Rizi M.S., Torkashvand S. (2021). A study on usability and design parameters in face mask: Concept design of UVW face mask for COVID-19 protection. Hum. Factors Ergon. Manuf. Serv. Ind..

[34] Seo S.K., Lee H.-K. (2021). The effect of face mask design on user’s face mask recognition and purchase behavior. J. Korea Des. Forum.

[35] Palcu J., Schreier M., Janiszewski C. (2022). Facial mask personalization encourages facial mask wearing in times of COVID-19. Sci. Rep..

[36] Lee M., You M. (2020). Psychological and behavioral responses in South Korea during the early stages of coronavirus disease 2019 (COVID-19). Int. J. Environ. Res. Public Health.

[37] Shin N., Lee K., Kang Y. (2021). A survey study of compliance with mask-wearing to prevent coronavirus infections among korean adults. J. Korean Acad. Fundam. Nurs..

[38] Asri A., Asri V., Renerte B., Föllmi-Heusi F., Leuppi J.D., Muser J., Nüesch R., Schuler D., Fischbacher U. (2021). Wearing a mask—For yourself or for others? Behavioral correlates of mask wearing among COVID-19 frontline workers. PLoS ONE.

[39] Lee E.H., Lee S.W., Moon S.Y., Son J. (2021). Performance Evaluation of Commercially Available Masks in Korea for Filtering Airborne Droplets Containing Bacteria. Int. J. Env. Res. Public Health.

[40] Kim M.-C., Bae S., Kim J.Y., Park S.Y., Lim J.S., Sung M., Kim S.-H. (2020). Effectiveness of surgical, KF94, and N95 respirator masks in blocking SARS-CoV-2: A controlled comparison in 7 patients. Infect. Dis..

[41] Chang H., Yim S.V. (2022). Mask in the era of COVID-19: Medical masks and the ecosystem of masks. Comp. Korean Stud..

[42] Kim M.-N. (2020). What type of face mask is appropriate for everyone-mask-wearing policy amidst COVID-19 pandemic?. J. Korean Med. Sci..

[43] Jung M.H., Mo Y., Park H.S. (2020). Factors on COVID-19 mask-wearing behavior. J. Humanit. Soc. Sci. (HSS21).

[44] Ha K.M. (2022). Changes in awareness on face mask use in Korea. Public Health Nurs..

[45] Koo M.J., Lee H.K. (2021). Strap design for mask due to COVID-19. J. Korean Soc. Des. Cult..

[46] Jung J.-Y., Lee J.-Y. (2021). Comparison of fit factor and total inward leakage of face masks: Exploratory evaluation by mask designs and face panels. J. Korean Soc. Living Environ. Syst..

[47] Kwon J., Lim G., Kim S.-H., Shin H.-J., Lee J.-Y. (2020). Risk Awaremess to COVID-19 and wear behavior of protective masks between adults and adolescent living in Seoul and Gyunggi Province. Korean J. Community Living Sci..

[48] Chao F.-L. (2020). Face mask designs following novel Coronavirus. J. Public Health Res..

[49] Yoo M.A., Kim S.H., Han H.S., Byun J.W., Park K.H. (2022). The effects of wearing a face mask and of subsequent moisturizer use on the characteristics of sensitive skin. Ski. Res. Technol..

[50] Park S.R., Han J., Yeon Y.M., Kang N.Y., Kim E. (2021). Effect of face mask on skin characteristics changes during the COVID-19 pandemic. Ski. Res. Technol..

[51] Saunders G.H., Jackson I.R., Visram A.S. (2021). Impacts of face coverings on communication: An indirect impact of COVID-19. Int. J. Audiol..

[52] Giovanelli E., Valzolgher C., Gessa E., Todeschini M., Pavani F. (2021). Unmasking the difficulty of listening to talkers with masks: Lessons from the COVID-19 pandemic. i-Perception.

[53] Balamurali B., Enyi T., Clarke C.J., Harn S.Y., Chen J.-M. (2021). Acoustic Effect of Face Mask Design and Material Choice. Acoust. Aust..

[54] Rahne T., Fröhlich L., Plontke S., Wagner L. (2021). Influence of surgical and N95 face masks on speech perception and listening effort in noise. PLoS ONE.

[55] Sfakianaki A., Kafentzis G.P., Kiagiadaki D., Vlahavas G. Effect of face mask and noise on word recognition by children and adults. Proceedings of 12th International Conference of Experimental Linguistics.

[56] Choi Y.-J. (2021). Acoustical measurements of masks and the effects on the speech intelligibility in university classrooms. Appl. Acoust..

[57] Toscano J.C., Toscano C.M. (2021). Effects of face masks on speech recognition in multi-talker babble noise. PLoS ONE.

[58] Ribeiro V.V., Dassie-Leite A.P., Pereira E.C., Santos A.D.N., Martins P., de Alencar Irineu R. (2020). Effect of wearing a face mask on vocal self-perception during a pandemic. J. Voice.

[59] Grieco-Calub T.M. (2021). COVID-19, Face Masks, and Social Interaction: How a Global Pandemic Is Shining a Light on the Importance of Face-to-Face Communication. Perspect. ASHA Spec. Interest Groups.

[60] Krishna A., Rodrigues J., Mitschke V., Eder A.B. (2021). Self-reported mask-related worrying reduces relative avoidance bias toward unmasked faces in individuals with low Covid19 anxiety syndrome. Cogn. Res. Princ. Implic..

[61] Saint S.A., Moscovitch D.A. (2021). Effects of mask-wearing on social anxiety: An exploratory review. Anxiety Stress Coping.

[62] Schneider A.B., Leonard B. (2022). From anxiety to control: Mask-wearing, perceived marketplace influence, and emotional well-being during the COVID-19 pandemic. J. Consum. Aff..

[63] Gallup Inc. (2020). The Coronavirus: A Vast Scared Majority around the World.

[64] Rieger M. (2020). To wear or not to wear? Factors influencing wearing face masks in Germany during the COVID-19 pandemic. Soc. Health Behav..

[65] Mallinas S.R., Maner J.K., Plant E.A. (2021). What factors underlie attitudes regarding protective mask use during the COVID-19 pandemic?. Personal. Individ. Differ..

[66] Lee M.-S. (2020). Fragmentary thoughts about code of conduct and risk communication to prevent and control COVID-19 in Korea, 2020. Korean J. Health Educ. Promot..

[67] Kwon M., Jang E.-M., Yang W. (2022). Mask-Wearing Perception of Preschool Children in Korea during the COVID-19 Pandemic: A Cross-Sectional Study. Int. J. Environ. Res. Public Health.

[68] Barceló J., Sheen G.C.-H. (2020). Voluntary adoption of social welfare-enhancing behavior: Mask-wearing in Spain during the COVID-19 outbreak. PLoS ONE.

[69] Firouzbakht M., Omidvar S., Firouzbakht S., Asadi-Amoli A. (2021). COVID-19 preventive behaviors and influencing factors in the Iranian population; a web-based survey. BMC Public Health.

[70] Knotek E.S., Schoenle R.S., Dietrich A.M., Müller G.J., Myrseth K.O.R., Weber M. (2020). Consumers and COVID-19: Survey results on mask-wearing behaviors and beliefs. Econ. Comment..

[71] Haischer M.H., Beilfuss R., Hart M.R., Opielinski L., Wrucke D., Zirgaitis G., Uhrich T.D., Hunter S.K. (2020). Who is wearing a mask? Gender-, age-, and location-related differences during the COVID-19 pandemic. PLoS ONE.

[72] Hearne B.N., Niño M.D. (2022). Understanding how race, ethnicity, and gender shape mask-wearing adherence during the COVID-19 pandemic: Evidence from the COVID impact survey. J. Racial Ethn. Health Disparities.

